# Measurable residual disease in chronic lymphocytic leukemia

**DOI:** 10.3389/fonc.2023.1112616

**Published:** 2023-02-14

**Authors:** Giulia Benintende, Federico Pozzo, Idanna Innocenti, Francesco Autore, Alberto Fresa, Giovanni D’Arena, Valter Gattei, Luca Laurenti

**Affiliations:** ^1^ Sezione di Ematologia, Dipartimento di Scienze Radiologiche ed Ematologiche, Università Cattolica del Sacro Cuore, Rome, Italy; ^2^ Clinical and Experimental Onco-Hematology Unit, Centro di Riferimento Oncologico di Aviano (CRO) Istituto di Ricovero e Cura a Carattere Scientifico (IRCCS), Aviano, Italy; ^3^ Dipartimento di Diagnostica per Immagini, Radioterapia Oncologica ed Ematologia, Fondazione Policlinico Universitario A. Gemelli Istituto di Ricovero e Cura a Carattere Scientifico (IRCCS), Rome, Italy; ^4^ “San Luca” Hospital, Azienda Sanitaria Locale (ASL) Salerno, Salerno, Italy

**Keywords:** measurable residual disease, chronic lymphocytic leukemia, flow cytometry, ASO-PCR, next generation sequencing, surrogate endpoint

## Abstract

Measurable residual disease (MRD) is defined as the presence of residual cancer cells after treatment in patients with clinically undetectable disease, who would otherwise be considered in complete remission. It is a highly sensitive parameter which indicates the disease burden and predicts survival in this setting of patients. In recent years, MRD has gained a role in many hematological malignancies as a surrogate endpoint for clinical trials: undetectable MRD has been correlated to longer progression free survival (PFS) and overall survival (OS). New drugs and combinations have been developed with the aim to achieve MRD negativity, which would indicate favorable prognosis. Different methods to measure MRD have also been devised, which include flow cytometry, polymerase chain reaction (PCR) and next generation sequencing (NGS), with different sensitivity and accuracy in evaluating deep remission after treatment. In this review, we will analyze the current recommendations for the detection of MRD, with particular focus on its role in Chronic Lymphocytic Leukemia (CLL), as well as the different detection methods. Moreover, we will discuss the results of clinical trials and the role of MRD in new therapeutic schemes with inhibitors and monoclonal antibodies. MRD is not currently used in the clinical practice to evaluate response to treatment, due to technical and economical limitations, but it’s gaining more and more interest in trials settings, especially since the introduction of venetoclax. The use of MRD in trials will likely be followed by a broader practical application in the future. The aim of this work is to provide a reader-friendly summary of the state of art in the field, as MRD will soon become an accessible tool to evaluate our patients, predict their survival and guide physician’s therapeutic choices and preferences.

## 1 Introduction

The use of Measurable Residual Disease (MRD) is extensive in acute myeloid diseases and other conditions where therapy has a curative objective. Oppositely, it has a controversial role in Chronic Lymphocytic Leukemia (CLL), which has changed over the years. Recently, MRD in CLL has raised interest again, thanks to the advent of target therapies which induce deep molecular response, such as the BCL-2 inhibitor venetoclax.

Undetectable MRD has been defined by the international workshop on CLL (iwCLL) and ERIC as the presence of <1 CLL cell per 10.000 leukocytes (1, 2). Standard staging methods with cytology can detect the presence of one CLL cell in up to a maximum of 100 leukocytes (3), therefore they are much less accurate than MRD in defining the burden of disease at the end of a treatment. According to the iwCLL criteria, we can consider a patient in complete remission (CR) when he/she presents with (i) less than 4x10^9^ lymphocytes/liter, more than 1.5x10^9^ neutrophils/liter, more than 100x10^9^ platelets/liter and more than 11.0 grams/deciliter hemoglobin level in peripheral blood; (ii) absence of lymphadenopathy >1.5 cm and splenomegaly or hepatomegaly at physical examination; (iii) absence of constitutional symptoms (1). Nevertheless, the clinical assessment alone is not considered accurate enough in the era of molecular biology and personalized therapy: thus, the need for a deeper definition of CR is emerging in clinical trials and will likely guide treatment choices in the clinical practice in the next future (4). Starting from the awareness that disease relapse comes from the expansion of any residual clone after therapy, we can easily get to the conclusion that the larger is the number of persistent clones, the earlier will relapse occur. As a matter of fact, even the smallest amount of residual leukemic cells can lead to relapse over time, when allowed to expand in the treatment free interval, if only looking at the clinical outcome of the previous treatment.

Given that clinical parameters correlate and can predict the PFS of those patients, a more powerful tool to predict such outcome is the highly sensitive detection of MRD, which is able to recognize very small amounts of residual clones in both peripheral blood (PB) and bone marrow (BM) (5). Nevertheless, determining MRD is more costly and technically difficult than clinical assessment, which explains why it is not yet recommended by the current guidelines and not routinely used in the clinical practice.

In the era of targeted therapies, monoclonal antibodies and combinations of such, the deepening of the response to treatment measured by MRD is considered an endpoint to establish the superiority of a therapeutic approach over another (6). To note, different treatment platforms obtain different MRD levels. The old chemo-immunotherapy regimen with Fludarabine-Cyclophosphamide-Rituximab (FCR) induces a long-lasting CR, at times accompanied by MRD negativity, which of course represents the most important predictor of survival (4). On the other hand, new targeted therapies obtain a heterogeneous variety of responses. Bruton tyrosine kinase receptor (BTK) inhibitors, including ibrutinib and acalabrutinib, obtain a rapid nodal reduction and increase of the hemoglobin and platelets levels, but are not able to reach MRD at any time, rather they induce partial remission (PR) which needs continuative administration of therapy, until relapse or toxicity, to maintain such response (4). Contrarily, the BCL2 inhibitor venetoclax, in combination with anti-CD20 antibody, has shown durable MRD negativity and a promising long-lasting progression-free survival in relapsed and refractory patients (7). Furthermore, the combination of BTK and BCL2 inhibitors (ibrutinib and venetoclax) achieves even deeper MRD negativity, and it has a favorable prognostic profile in terms of PFS, but it is now available only in few clinical trials (8).

In this review, we will go through all the laboratory methods that allow the definition of MRD with different rates of sensitivity as well as different costs and technical requirements. We will try to summarize the state of art in the detection of MRD on PB rather than on BM. We will also focus on the impact of MRD on both traditional and emerging therapeutic approaches, and its relevance to tailor the treatment based on patients’ age, clinical status, and future perspectives.

We strongly believe that MRD has a crucial impact on the definition of personalized therapeutic strategies, as new clinical trials involve the detection of MRD to delineate next steps of patients’ management. Therefore, it is important for any clinician to have a clear idea of the meaning of MRD detection from a technical point of view, but more relevantly, as a tool that will possibly be introduced in real life to guide and refine treatment choices.

## 2 MRD detection methods

Thanks to the technical advances of the last years, different methods to determine the burden of residual disease in CLL patients after treatment are available. At the same time, the lack of standardized guidelines makes the comparison between different clinical trials hard, due to the heterogenicity of techniques used and their sensitivity in detecting persistent clones (2). We will try to display the currently available options according to updated recommendations. **Table 1** summarizes the difference between the three methods in terms of sensitivity, target, and standardization.

**Table 1:** Summary table comparing the sensitivity of the three laboratory methods (flow-cytometry, PCR and NGS) in terms of sensitivity, target, and standardization.

	*Flow cytometry*	*ASO-PCR*	*NGS*
***Sensitivity***	MRD5	MRD6	MRD5
***Target***	CD19, CD20, CD5, CD43, CD79b and CD81	Ig hypervariable region	CDR3 sequence of the Ig
***Standardization***	ERIC 2016	none	none

### 2.1 Flow cytometry

Multiparametric flow cytometry allows automated phenotyping of cells with fluorescently labelled antibodies (9). Panels of antibodies linked to different fluorochromes can identify a specific CLL phenotype, characterized by expression of certain surface antigens.

The first attempts to measure minimal disease evaluated the clonality by immunoglobulin light chain (κ or λ) restriction on a CD19/CD5 co-expressing population (10). This approach, virtually applicable to all CLL cases, later demonstrated a low sensitivity and was deemed unsuitable for predicting response status according to later iwCLL/NCI criteria or identifying cases with no detectable MRD (11).

**Figure 1:**
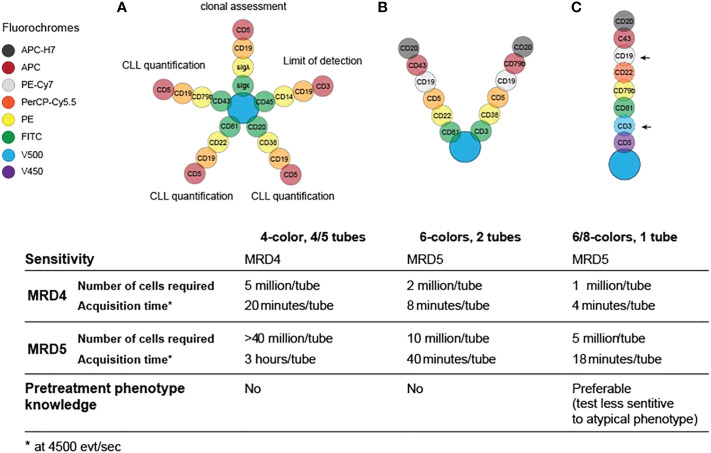
Evolution and requirements for flow cytometry panels for MRD testing in CLL in 2007 **(A)**, 2013 **(B)** and 2016 **(C)**.

The standardized cytofluorimetric approach for the detection of MRD dates to 2007, but it still gives valuable information to assess the presence of residual cells on PB (2). Cell preparation was performed by a whole-blood lysis method with or without fixatives such as ammonium chloride or FACSLyse, to allow quantitative enumeration of CLL cells. The antibodies used to detect MRD were against CD19, CD5, CD20, CD38, CD22, CD81, CD43, CD79b, combined in four different four-color tubes: one clonality tube (CD19, CD5, surface light chains κ or λ), one limit of detection tube (CD19, CD3, CD45, CD14) and three tubes dedicated to MRD enumeration (2) (**Figure 1A**).

This first protocol was suitable for the detection of 1 cell in 10.000 lymphocytes in PB within an adequate sample of 1 to 2 million cells, thus a sensitivity of 0.01%/10^-4^, also termed MRD4 according to Wierda et al (6). Even though this four-colors set of antibodies showed good performance and multiple standardization measures were adopted (2), there was a significant inter-laboratory variability and the MRD determination was still highly operator-dependent. Moreover, this approach needed four/five tubes, which further increased the risk of procedural errors.

With the evolution of flow cytometry instruments, more parameters became readily available and the MRD panel was improved to two 6-color tubes (CD19/CD5/CD20/CD3/CD38/CD79b and CD19/CD5/CD20/CD81/CD22/CD43; **Figure 1B**) (11). This approach reduced the amount of time and sample required for MRD enumeration and reached the ability to quantitatively detect residual disease in the 0.001–0.01% (MRD4-MRD5) range.

**Figure 2:**
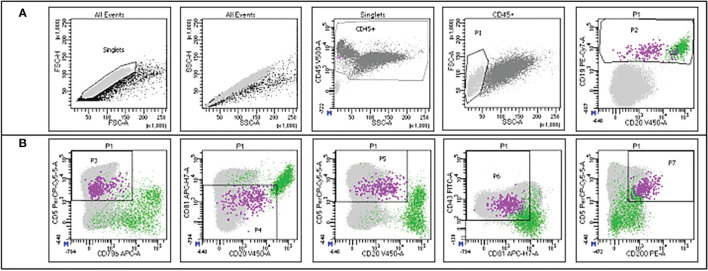
Example of the gating strategy employed for MRD detection in the flow cytometry panel. The green dots represent normal B cells, while the violet dots represent CLL cells. The gates are set up hierarchically. **(A)** Singlets are selected on FSC-H/FSC-A; the same population is refined on SSC-H/SSC-A; leukocytes are selected through the CD45 staining on CD45/SSC; mononuclear cells are selected on FSC/SSC (P1) and B cells are selected on CD19/CD20 (P2). **(B)** CLL cells are further characterized according to CD5/CD79b (P3), CD81/CD20 (P4), CD5/CD20 (P5), CD43/CD81 (P6) and CD5/CD200 (P7) expression. Courtesy of Prof. Giovanni D´Arena and Dr. Antonella Aiello.

In 2016, the European Research Initiative on CLL (ERIC) further validated a standardized flow cytometry approach to reliably detect CLL clones up to the level of 0.001% (MRD5) on a single tube: this assay includes a core panel of six markers, namely CD19, CD20, CD5, CD43, CD79b and CD81, as summarized in **Figure 1C**. Although the initial panel was designed with 8 colors, including CD22 and CD3, these markers were ultimately considered not essential, and the latter was deemed informative only if a very high accuracy (<10^-5^) was necessary (**Figure 1C**, arrows). This system was designed to work independently from reagents and laboratory equipment (e.g., by processing the ratio of median fluorescence intensity of the positive signal over a negative signal, rather than raw fluorescence intensities), and could be validated locally, in different laboratories, using normal PB. To confirm the reliability of this 6-color, 1-tube method, a parallel analysis of high-throughput sequencing with ClonoSEQ assay was performed and showed good concordance with flow cytometry results at the MRD4 level, which represents the MRD threshold defined by the iwCLL guidelines in 2008 (12). Nevertheless, this method demonstrated to provide good qualitative results up to a detection limit of 1 in a million (10^-6^) (13). The only significant drawback of this setup is that can be insensitive in presence of atypical phenotypes, therefore the knowledge of the pre-treatment phenotype is advisable (11). An example of a flow cytometry panel for MRD detection can be visualized in **Figure 2**.

Flow cytometry has the advantage to be a rapid method which works for most of typical CLL cases with very good sensitivity. Moreover, these instruments are widespread in most diagnostic laboratories, and they are operator friendly and easy to run, making this technique the preferred choice of most clinical investigators. On the other hand, the disadvantage is that samples must be processed within 48h and in any case not later than 72h, so fresh blood preparations are needed, and no cell storage can be performed (2, 14).

Over time, several integrations and extensions have been proposed, to increase the sensitivity and limit of detection of the ERIC 6-color panel, also incorporating novel markers such as ROR1, CD200, CD160 (15–17) [also reviewed in D’Arena et al. (18)].

Innovative next-generation flow cytometry methods, capable of recording tens of millions of events and coupled with advanced analysis software, may potentially allow the reach of MRD6 in the next future (19). However, these systems will likely require significant hardware and software capabilities, initially limiting the application of these innovative technologies to few specialized laboratories.

### 2.2 Polymerase chain reaction-based methods: Allele-specific and digital PCR

**Figure 3:**
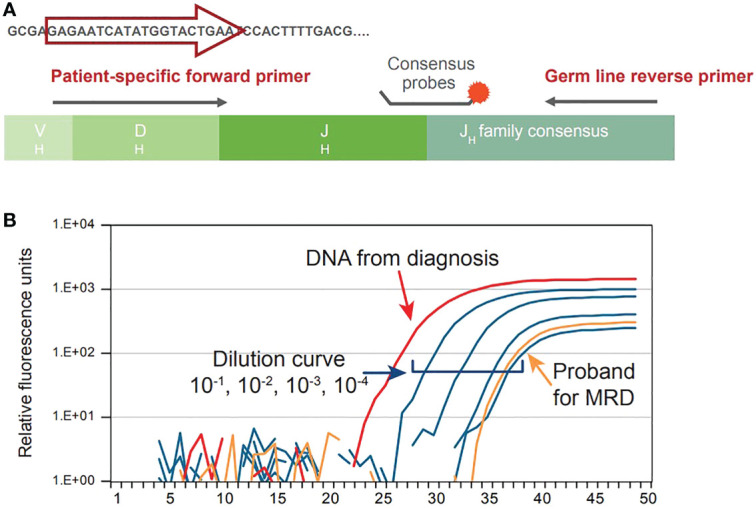
Graphical representation of ASO-PCR procedure **(A)** and output **(B)**.

Given that CLL is caused by aberrant proliferation of a specific B-cell population, each B-cell clone can be identified on a genetic level based on its uniquely rearranged immunoglobulin (Ig) genes within hypervariable regions. These regions are a unique characteristic of the leukemic cell; therefore, allele specific oligonucleotide (ASO) PCR takes advantage of the patient-specific Ig gene rearrangement to identify CLL clones and detect MRD. In this method, ASO primers matching the hypervariable region of each leukemic cell are used with reverse consensus JH germline primers and a fluorescent hydrolysis probe annealing to a downstream family specific JH region on a real-time thermal cycler. A graphical representation of the procedure can be visualized in **Figure 3A**, while the output of this procedure is shown in **Figure 3B**. Calculation of the MRD level is based on comparative analysis between follow-up samples and standard cells in ASO from polyclonal DNA, normalized to albumin PCRs as internal control (20). Guidelines for the interpretation of ASO-PCR results are available and attempt to standardize the results across different laboratories. Application of such guidelines and strict quality control assure the comparability of results obtained from different clinical trials (21).

The sensitivity of this method has been attested between 10^-4^ and 10^-5^. Moreover, this method can be applied to frozen samples, which can be stored for a long time, and does not require processing of fresh samples (20). On the other hand, this method is intrinsically dependent on the amount of amplified DNA: in this regard, reliable clonotype identification requires that at least 3-5 copies of target DNA should be present within the sample; an adequate amplification would therefore need at least 100 nanograms of DNA for MRD4, 1 microgram for MRD5, 10 micrograms for MRD6. Furthermore, since ASO-PCR is tailored to be patient-specific, a representative pathological sample is required to set-up of the method, for positive controls in subsequent testing, as well as for the standard dilution curve for determining limit of detection and quantitative range in each experimental session; running out of such material would impair the quantitative power of ASO-PCR, which could be used only for qualitative assessment of MRD. Overall, this method can be very powerful but requires an expertise which is not available in any laboratory, thus rendering its use more difficult in the routine clinical practice.

An improvement of PCR-based detection is digital PCR [see also Dogliotti et al (22)], in which single DNA molecules are encapsulated in a confined space and amplified in presence of a fluorescent reporter (intercalating dye or hydrolysis probe); the resulting amplification will theoretically be either positive, if target DNA is present, or negative, hence the term “digital”. The main advantage is that this is a quantitative technique, as it does not require a standard curve (hence does not possess a quantitative range) and is independent of reaction efficiency. A sensitivity up to MRD5, and possibly MRD6, is reached when the amplification is selective for known specific alterations, as *BCL2*-IgH translocation in Follicular Lymphoma, or *MYD88* L265P Waldenström Macroglobulinema (23, 24). This is not however the case of CLL, where a single lesion, acting as a tracker, is not present; in this case, patient-specific lesions may be exploited to monitor the disease’s trajectory, but the emergence of novel clones without the monitored mutations must be taken into consideration.

### 2.3 Next generation sequencing

Ultra-deep next-generation sequencing (NGS) has emerged in recent years as an important diagnostic tool for the quantification of tumor burden, since many patients with undetectable MRD at flow cytometry and/or ASO-PCR, which both have a 10^-4^ sensitivity, as recommended by the iwCLL guidelines, relapse after few years, especially if their disease is characterized by high-risk molecular features such as unmutated IGHV. NGS can identify residual cells by amplification of all VDJ sequences from a single DNA sample (25, 26); the method requires previous knowledge of the specific CDR3 sequence of the immunoglobulin expressed by the pathological clone, which the investigator can later search for in MRD samples. Compared to ASO-PCR, NGS has the advantage that the amplification does not require patient-specific primers but is only dependent on the amount of loaded DNA, for which the MRD5 target (1 microgram of input DNA) is generally achievable (27). Therefore, undetectable MRD by NGS represents nowadays the most reliable predictor of survival in CLL patients. The main drawback is that NGS is not widely available, and economically viable only for centers facing significant volumes of testing; on the other hand, these centers would be equipped with adequate instrumentation and automate most of the analyses, significantly reducing the raw costs of a single test and the handling time and building up the necessary expertise to analyze NGS data efficiently. Therefore, at present, one of the optimal contexts for NGS in MRD evaluation resides within clinical trials, which are likely to centralize the most expensive analyses, thus also guaranteeing some degree of standardization. The absence of highly standardized commercial methods limits the applicability of NGS in the routine clinical practice and this is probably why, for now, it is not mentioned by the iwCLL recommendations (1).

The current landscape of technologies for MRD detection is quickly approaching a steady MRD5 detection limit, and the final choice of method is ultimately dependent on each laboratory’s set up. Flow cytometry may be most suited for laboratories with an established cytometry facility, standardized instruments and trained personnel; however, it has a short “vein-to-brain” turnaround time, and the result can be produced within a few hours. Oppositely, ASO-PCR is a method that can be implemented in most molecular laboratories as it does not rely on a particularly advanced equipment if applied through real-time PCR, whereas digital PCR is more limited to specialized centers; NGS is equally, if not more, elective to specialized facilities, however it has the significant advantage that the investigator can search for the CDR3 sequence of the pathological clone directly within the sequencing output, providing higher sensitivity and specificity (28). Overall, these techniques may represent different but complementary tools for a comprehensive MRD detection, providing molecular detection where the cell phenotype may vary (for example CD20 expression after therapy with rituximab) or, vice versa, rely on a stable phenotypic marker in presence of ongoing somatic hypermutation and intra-clonal diversification of IGHV genes which may hamper patient specific CDR3 recognition.

## 3 MRD detection in peripheral blood vs bone marrow

CLL is characterized by the accumulation of leukemic cells in PB, BM and lymphoid tissues as spleen, liver, and lymph nodes (29). Therefore, the presence of leukemic cells in different tissues claims for clarification of the best candidate samples to determine MRD. MRD status is strongly prognostic for PFS and OS both in PB and BM of CLL patients after treatment (30). Nevertheless, the multi-compartment nature of CLL suggests the possibility of discordant MRD results on different tissues; thus, the sampling site may affect the prognostic ability of this parameter, and the choice depends on many factors such as timing of the sampling and treatment status. In general, it has been demonstrated that concordance between PB and BM MRD status is ~85% at the 10^-4^ threshold (6). For the anti-CD20 monoclonal antibody Rituximab, the concordance lowers to 79%: the sensitivity of MRD detection in BM is higher than that of PB, with added value for predicting prognosis or treatment effects (31). Nevertheless, the collection of BM samples is invasive and painful for patients, so it cannot be used in routine follow up; therefore, samples from PB are commonly used instead.

## 4 Clinical significance of MRD in the era of targeted therapies

**Figure 4:**
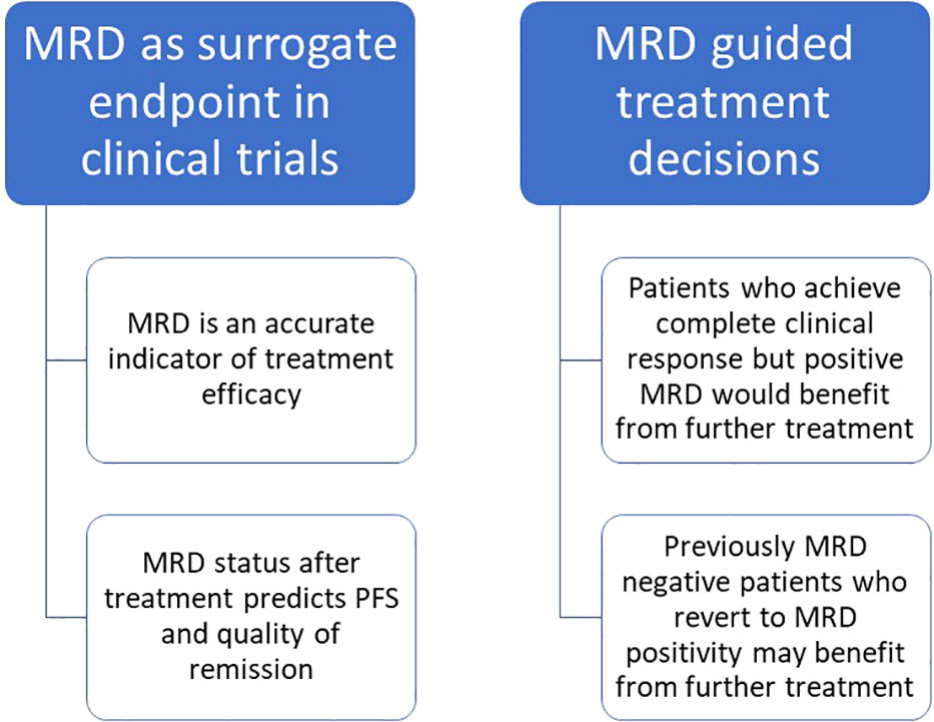
Role of MRD in the clinical practice and potential use in routine management of CLL patients.

Some history of CLL treatment may be helpful to understand the role of MRD in the current and future clinical practice. Before 1990, any CLL treatment aimed at palliation: they included alkylating agents as chlorambucil or purine analogues as fludarabine in monotherapy. The advent of combination treatments including both fludarabine and cyclophosphamide improved the survival outcomes and response rates, even if the real revolution happened in 2010 with the introduction of the anti-CD20 monoclonal antibody rituximab, which combined to fludarabine and cyclophosphamide (FCR) gained great results in terms of survival (32). Around 2014, the combination of obinutuzumab and chlorambucil was devised for elderly and frail patients (33). In the same year, the anti-BTK inhibitor ibrutinib (34) opened the era to targeted therapies, followed later by the anti-BCL2 inhibitor venetoclax (35). The advances of CLL treatment led to improvement of the long-term outcomes in terms of survival and depth of response, which is why nowadays MRD became a valuable instrument in the post-treatment evaluation of patients (36). MRD can have a role as surrogate primary endpoint in clinical trials, since it is an accurate indicator of treatment efficacy which predicts PFS (36). On the other hand, it can be used as a determinant of future treatment choices since patients who do not achieve MRD-negativity after treatment can benefit from further treatment or new molecules to achieve a deeper remission and prolong PFS (5, 37). The timing of MRD assessment can vary depending on the duration of the treatment and on the use of continuous or fixed time regiments, for which MRD is usually measured at the end of the treatment. **Figure 4** summarizes the role of MRD in the clinical practice.

### 4.1 MRD assessment after chemo-immunotherapy

The evaluation of MRD with the recommended sensitivity of 10^-4^ can predict survival of naïve patients undergoing first line treatment with chemo-immunotherapy. Several studies investigated the results in term of MRD negativity after different combination therapies, all proving that MRD is an independent predictor of survival (30).

Lamanna et al. investigated the prevalence of MRD negativity in patients treated with sequential fludarabine, high dose cyclophosphamide and rituximab as first line, and found 56% prevalence of MRD negativity by flow cytometry and 33% by PCR in PB (38).

The German group established the addition of rituximab to fludarabine and cyclophosphamide in 2010, through the CLL8 trial which achieved great results compared to the past (32). Subsequently, Boettcher et al. analyzed the clinical significance of flow cytometric MRD between the arms of the CLL8 trial, quantifying MRD in both PB and BM and categorizing patients into low/undetectable (<10^-4^), intermediate (10^-4^-10^-2^) and high (>10^-2^) level of MRD detected on PB. PFS was 68.7% for the low MRD group and 40.5% for the intermediate and high MRD groups. The results of this analysis showed that the level of MRD after FCR is predictive of both OS and PFS, which validates the use of MRD as a marker to assess the efficacy of such treatment (39).

The German group also investigated the prevalence of MRD-negativity in previously untreated patients undergoing treatment with bendamustine and rituximab (BR): 57.8% of them obtained MRD negativity below 10^-4^ in PB, which was associated to longer event-free survival compared to patients who did not achieve such deep remissions (40).

The CLL11 trial by Goede et al. investigated the prevalence of MRD negativity evaluated by PCR on PB, which was 37.7%, and BM, which was 19.5%. Also, in this case, MRD negativity was predictive of improved event-free survival (33).

### 4.2 MRD assessment after BTK inhibitors

The long-lasting experience with chemo-immunotherapy led to the awareness of the importance of MRD in CLL as predictor of treatment outcome and survival. The introduction of ibrutinib has opened the way to target therapies, initially as second line in relapsing CLL (34) and then as first line in previously untreated CLL patients (41). Its biological mechanism of action involves binding of the ibrutinib molecule to the ATP active site of the BTK which blocks the constitutionally activated BCR signaling involved in cells survival and expansion (42). Despite the encouraging results in terms of survival, ibrutinib is characterized by the maintenance of MRD positivity on the long term, thus requiring continuous therapy until either progression of the disease or toxicity of the drug. After its introduction, given the excellent clinical response but poor result in terms of MRD negativity, the role of MRD as predictor of survival was questioned (36). According to Ahn et al., MRD negativity was achieved only by 10.2% of both treatment naïve and relapsed/refractory patients after 5 years of continuous ibrutinib administration, but this result was surprisingly correlated to great outcomes in terms of PFS (74.4% of patients) and OS (85.3% of patients). The CR rate was 37.5% in the low MRD group and 21.3% in the high MRD group, but PFS was not statistically different between the 2 groups. Therefore, when it came to treatment with ibrutinib, MRD was not predictive of poor event-free survival in patients treated with monotherapy (43).

The combination of ibrutinib and rituximab did not obtain better results compared to monotherapy in terms of MRD negativity tested in PB at 12 months (8.3% vs 59.2% in patients treated with FCR): nevertheless, PFS was 65% and OS was 83%, lower with ibrutinib and rituximab than with chemo-immunotherapy, so again MRD was not predictive of lower event-free survival (44).

The ILLUMINATE trial investigated the efficacy of the combination of ibrutinib and obinutuzumab compared to chlorambucil and obinutuzumab. This study obtained the best result in terms of MRD negativity for ibrutinib, which was 30% in PB (vs 20% in the Chl-Obinu group) and 20% in BM (vs 17% in the Chl-Obinu group).

Finally, the HELIOS trial investigated the combination of ibrutinib with BR compared to BR alone. The rate of MRD negativity given by the combination of ibrutinib and BR was 26.3% (45), so also in this case it was significantly higher than for ibrutinib monotherapy and combination of ibrutinib and rituximab.

**Figure 5:**
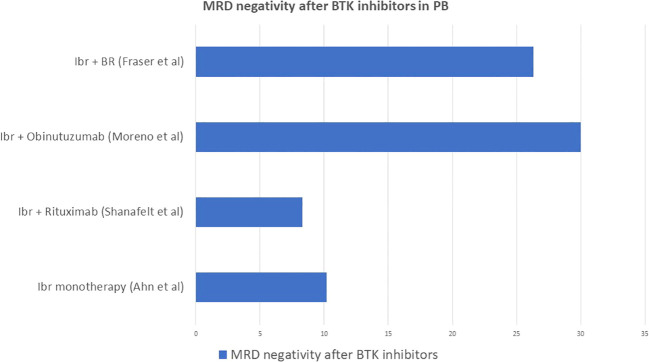
Rate of MRD negativity obtained by ibrutinib monotherapy, ibrutinib combined with anti CD20 monoclonal antibodies or with BR.

**Figure 5** summarizes the rate of MRD negativity obtained by ibrutinib monotherapy (43), ibrutinib combined with anti CD20 monoclonal antibodies (44, 46) or with BR (45).

### 4.3 MRD assessment after BCL2 inhibitors

The anti-BCL2 inhibitor venetoclax, on the other hand, is characterized by higher rates of MRD negativity, also in this case defined as less than 10^-4^, in CLL. A pooled analysis of patients enrolled in different clinical trials, which we will report below, showed an overall 42% of confirmed undetectable MRD in either PB, BM or both. The median time to obtain MRD negativity was 18 months and 90% of patients obtained MRD negativity within 24 months, while no patient obtained MRD negativity after 24 months without dose escalation. Deletion of chromosome 17p correlated, as expected, to a lower probability to obtain MRD negativity and consequently to a higher rate of relapse. Of those who did not obtain MRD negativity, 78% patients developed progressive disease at a median time of 19 months, confirming that, also upon treatment with venetoclax, MRD is a strong predictor of event-free survival (47).

The efficacy of venetoclax monotherapy has been investigated in studies which included patients with heterogeneous chromosome 17p deletion and *TP53* status as well as previous exposure to BTK inhibitors (48). The M13-982 study investigated CLL patients with relapsed/refractory (R/R) disease and with 17p deletion, a small number of whom had previously received BTK inhibitors: 20% of the enrolled patients obtained MRD negativity in PB (35). The M14-032 study included CLL patients who previously failed treatment with BTK inhibitors, regardless of their mutation status: 42% of the enrolled patients obtained MRD negativity in PB. Moreover, 71% of patients who progressed after treatment with BTK inhibitors responded to venetoclax (49).

The combination of venetoclax and rituximab as fixed therapy for 24 months in relapsed/refractory CLL has been investigated by the MURANO trial, which compared it to the traditional chemo-immunotherapy with bendamustine and rituximab. This study showed an encouraging high rate of MRD negativity in PB at 9 months for the venetoclax and rituximab group (62.4%) compared to the BR counterpart (13.3%), which strictly correlated with a longer event-free survival (84.9% vs 34.8% at 2 years). Within the venetoclax-rituximab arm, patients with undetectable MRD achieved 85% PFS, while those with detectable MRD 65% (50).

The CLL14 trial investigated the efficacy of venetoclax and obinutzumab, as fixed therapy for 12 months, against chlorambucil and obinutuzumab in first line. This study reported a 76% of MRD negativity at end of treatment in the group who received venetoclax and obinutuzumab, which correlated to a longer PFS compared to the Chl-Obi group, with a 0.31 hazard ratio (CI 0.22-0.44) (51).

The CLL13 trial investigated the outcome of four different therapeutic schemes: CIT with FCR or BR, venetoclax plus rituximab, venetoclax plus obinutuzumab and venetoclax plus obinutuzumab and ibrutinib in first line for fit patients. This study reported 52% MRD negativity for FCR or BR, 57% for venetoclax plus rituximab, 86.5% for venetoclax plus obinutuzumab and 92.2% for venetoclax plus obinutuzumab and ibrutinib (52).

**Figure 6:**
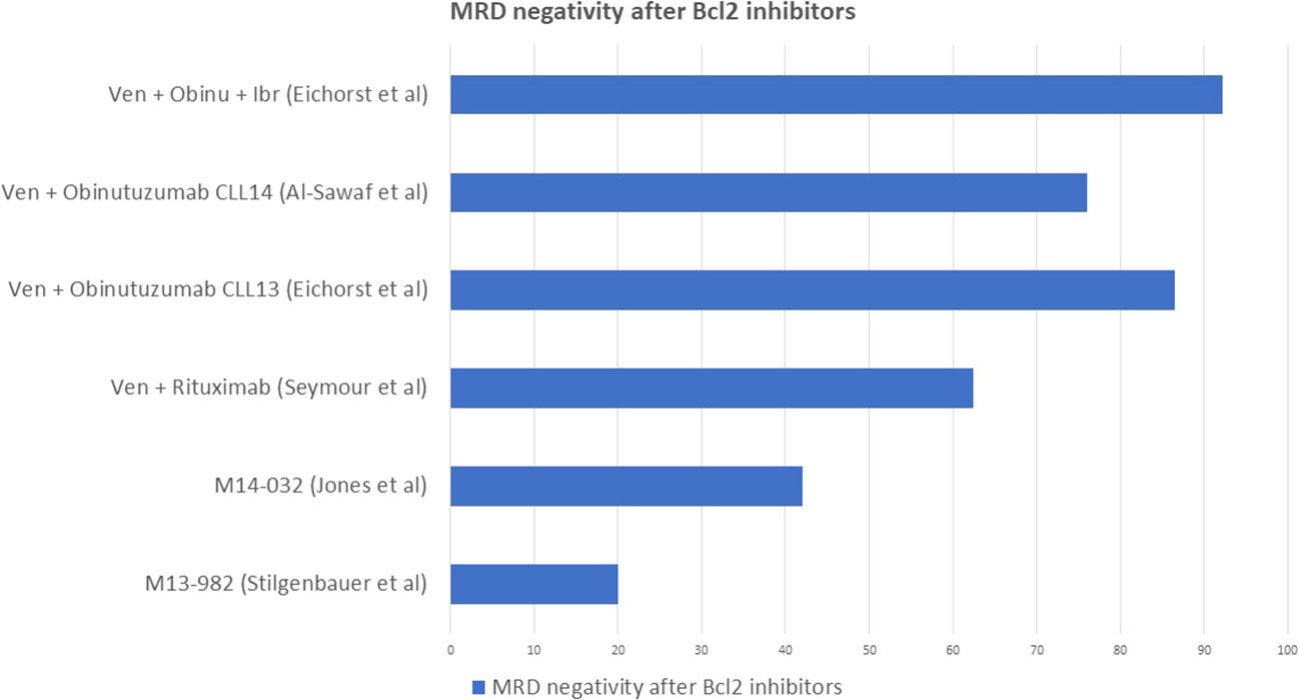
Rate of MRD negativity obtained by venetoclax monotherapy and venetoclax combined with anti CD20 monoclonal antibodies.

**Figure 6** summarizes the best rates of MRD negativity obtained by venetoclax monotherapy (35, 49), venetoclax combined with anti CD20 monoclonal antibodies (50, 51) and with ibrutinib (52) in different clinical trials.

### 4.4 New combinations and future perspectives

**Figure 7:**
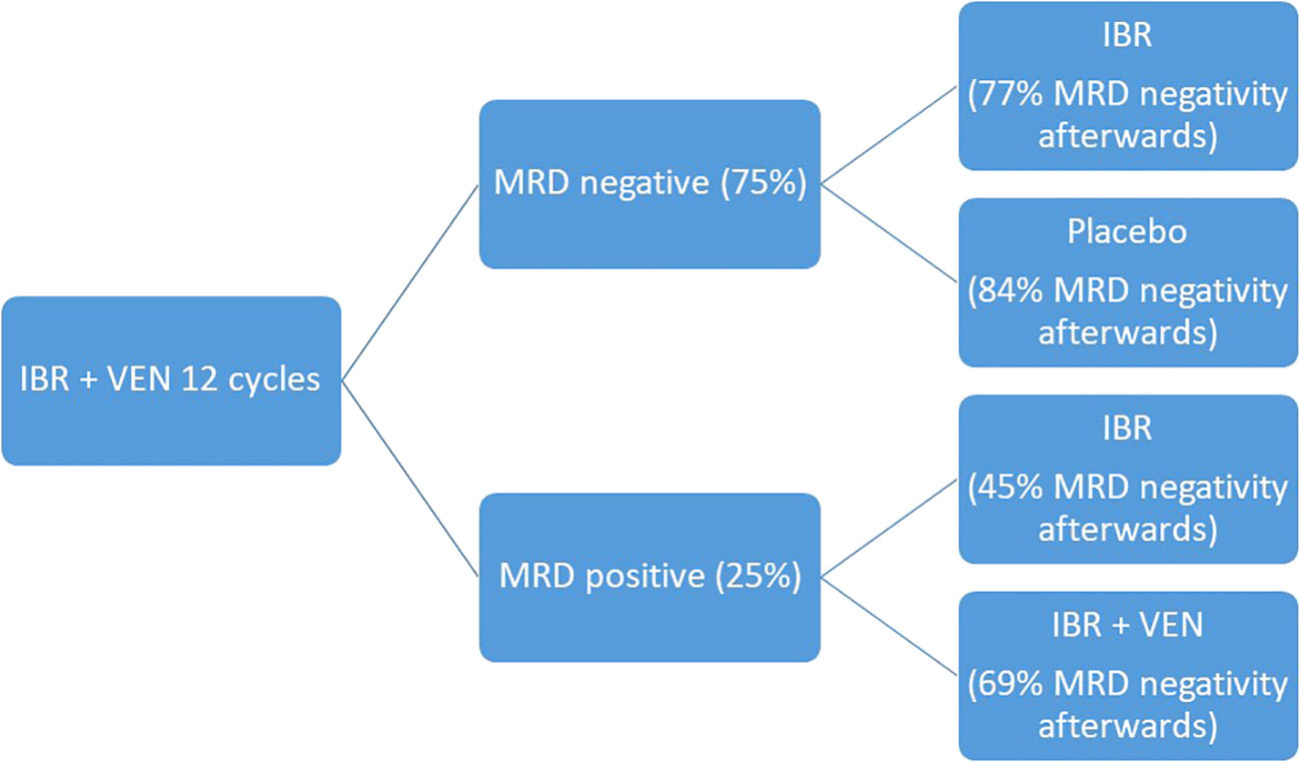
CAPTIVATE study design and results in terms of MRD negativity.

The development of target therapies for CLL and the spread of rapidly available MRD detection methods opened the way to a wider MRD use in clinical practice. New trials aim at more and more personalized therapies, where the treatment strategies can be designed based on patients’ epidemiological characteristics, molecular biology of the disease and MRD detection at end of treatment. The CAPTIVATE trial moves in this direction: it is a multicenter randomized phase II trial which studies the combination of ibrutinib and venetoclax in two cohorts, the MRD-guided and the fixed-duration cohort. For the sake of our topic, we will describe the study design for the MRD guided cohort: during the pre-randomization phase, patients received ibrutinib monotherapy for three cycles followed by ibrutinib and venetoclax for 12 cycles. At the end of the 12 cycles, MRD was tested, and patients were divided into MRD negative cohort, which was randomized to ibrutinib continuation or placebo, and MRD positive cohort, which was randomized to ibrutinib monotherapy or continuation of ibrutinib and venetoclax. At the end of the pre-randomization phase, 75% of patients obtained MRD negativity in PB and 68% in BM. After the randomized phase, in the MRD negative cohort, undetectable MRD in PB went from 100% to 84% for patients who received placebo and from 100% to 77% for patients who received ibrutinib. In the MRD positive cohort, undetectable MRD remained 45% for patients who received ibrutinib monotherapy while it went from 50% to 69% for those who received the combination of ibrutinib and venetoclax (53). Such encouraging results support the preclinical evidence of a synergistic effect of ibrutinib and venetoclax, which target the BTK and Bcl2 receptors at the same time resulting in higher cytotoxicity, and consequently deeper molecular response, compared to the two drugs alone (54). Comparable results in terms of MRD were also confirmed in the cohort treated with the fixed duration regimen, with achievement of deep molecular response which correlated to a favorable PFS (55). **Figure 7** summarizes the study design and results in terms of MRD negativity.

The importance of this trial is to open the way to a more and more tailored approach which address patient’s needs depending on the depth of remission they obtain, beyond the clinical characteristics. Other than the effectiveness of the two drugs and their combination, it is important to consider how this trial emphasizes the role of MRD in guiding physician’s choices on treatment management, which may reflect an upcoming practical application of MRD in the clinical practice.

## 5 Conclusion

Measurable residual disease has always gained interest in the field of hematology, but its importance is increasing steadily thanks to the advances in the treatment of CLL. Nevertheless, MRD does not currently have a defined role in the clinical practice. The three main laboratory methods used to detect MRD include flow cytometry (2), ASO-PCR (20) and NGS (26), and they all have a role in clinical trials- The cytometric method standardized by the ERIC guidelines (2, 11) appears to be the most accessible, in terms of feasibility and costs, in the clinical practice.

The development of chemo-immunotherapy combination platforms (FCR, BR, Chl-Obinu) back in the days, shed light on the possibility to deepen the molecular response of CLL and obtain a long-lasting event free survival (39). Surprisingly, the advent of ibrutinib discouraged the use of MRD as a surrogate endpoint for PFS, as long-lasting partial responses were obtained with continuative ibrutinib treatment regardless of persistent MRD negativity (36). Venetoclax monotherapy or in combination with anti-CD20 monoclonal antibodies restored the key role of MRD in clinical trials and validated its correlation to event free survival in patients treated with the BCL2 inhibitor (35, 47–51). Furthermore, the newest combination of ibrutinib and venetoclax, which already showed a synergistic effect in pre-clinical models, obtained even deeper molecular response, and explored the use of MRD itself to determine further steps of patients’ management (53–55).

In conclusion, MRD has a valuable role in defining remission at a more profound level compared to clinical assessment alone, and it can help guiding treatment choices to obtain a more durable event free survival, which is a turning point for such a chronic and incurable condition. For this reason, even if at present MRD is not part of routine evaluation of patients at the end of treatment in the clinical setting, it may gain a role in the next years and it may even be included in new guidelines, as part of the recommended steps to establish patients’ response and prognosis.

## Author contributions

GB and FP wrote the paper. VG, FP and GD’A provided the cytofluorimetric background and the related figures. VG and FP provided the ASO-PCR and NGS background. FP, II, AF, FA revised and corrected the manuscript. LL supervised and coordinated the work. All authors contributed to the article and approved the submitted version.

## References

[1] Hallek M Cheson BD Catovsky D Caligaris-Cappio F Dighiero G Döhner H . iwCLL guidelines for diagnosis, indications for treatment, response assessment, and supportive management of CLL. Blood (2018) 131(25):2745–60. doi: 10.1182/blood-2017-09-806398 29540348

[2] Rawstron AC Villamor N Ritgen M Böttcher S Ghia P Zehnder JL . International standardized approach for flow cytometric residual disease monitoring in chronic lymphocytic leukaemia. Leukemia (2007) 21(5):956–64. doi: 10.1038/sj.leu.2404584 17361231

[3] Guillaume N Alimardani G Capiod JC Claisse JF . [Relevance of cytological and immunophenotypical analysis for the diagnosis of b-cell chronic lymphocytic leukaemia]. Ann Biol Clin (Paris) (2002) 60(6):673–81. 12446231

[4] Thompson PA Wierda WG . Eliminating minimal residual disease as a therapeutic end point: working toward cure for patients with CLL. Blood (2016) 127(3):279–86. doi: 10.1182/blood-2015-08-634816 PMC4828075 26576865

[5] Böttcher S Hallek M Ritgen M Kneba M . The role of minimal residual disease measurements in the therapy for CLL. Hematol Oncol Clin North Am (2013) 27(2):267–88. doi: 10.1016/j.hoc.2013.01.005 23561473

[6] Wierda WG Rawstron A Cymbalista F Badoux X Rossi D Brown JR . Measurable residual disease in chronic lymphocytic leukemia: Expert review and consensus recommendations. Leukemia (2021) 35(11):3059–72. doi: 10.1038/s41375-021-01241-1 PMC8550962 34168283

[7] Seymour JF Kipps TJ Eichhorst BF D’Rozario J Owen CJ Assouline S . Enduring undetectable MRD and updated outcomes in relapsed/refractory CLL after fixed-duration venetoclax-rituximab. Blood (2022) 140(8):839–50. doi: 10.1182/blood.2021015014 PMC9412011 35605176

[8] Hillmen P Pitchford A Bloor A . The combination of ibrutinib plus venetoclax results in a high rate of MRD negativity in previously untreated CLL: The results of the planned interim analysis of the phase III NCRI FLAIR trial. Vienna: 2022 EHA Congress (2022).

[9] Salem DA Stetler-Stevenson M . Clinical flow-cytometric testing in chronic lymphocytic leukemia. Methods Mol Biol (2019) 2032:311–21. doi: 10.1007/978-1-4939-9650-6_17 PMC8276061 31522426

[10] Rawstron AC Kennedy B Evans PAS Davies FE Richards SJ Haynes AP . Quantitation of minimal disease levels in chronic lymphocytic leukemia using a sensitive flow cytometric assay improves the prediction of outcome and can be used to optimize therapy. Blood (2001) 98(1):29–35. doi: 10.1182/blood.V98.1.29 11418459

[11] Rawstron AC Böttcher S Letestu R Villamor N Fazi C Kartsios H . Improving efficiency and sensitivity: European research initiative in CLL (ERIC) update on the international harmonised approach for flow cytometric residual disease monitoring in CLL. Leukemia (2013) 27(1):142–9. doi: 10.1038/leu.2012.216 23041722

[12] Hallek M Cheson BD Catovsky D Caligaris-Cappio F Dighiero G Döhner H . Guidelines for the diagnosis and treatment of chronic lymphocytic leukemia: a report from the international workshop on chronic lymphocytic leukemia updating the national cancer institute–working group 1996 guidelines. Blood (2008) 111(12):5446–56. doi: 10.1182/blood-2007-06-093906 PMC2972576 18216293

[13] Rawstron AC Fazi C Agathangelidis A Villamor N Letestu R Nomdedeu J . A complementary role of multiparameter flow cytometry and high-throughput sequencing for minimal residual disease detection in chronic lymphocytic leukemia: an European research initiative on CLL study. Leukemia (2016) 30(4):929–36. doi: 10.1038/leu.2015.313 PMC4832072 26639181

[14] Rawstron AC Kreuzer KA Soosapilla A Spacek M Stehlikova O Gambell P . Reproducible diagnosis of chronic lymphocytic leukemia by flow cytometry: An European research initiative on CLL (ERIC) & European society for clinical cell analysis (ESCCA) harmonisation project. Cytom B Clin Cytom (2018) 94(1):121–8. doi: 10.1002/cyto.b.21595 PMC5817234 29024461

[15] Bento L Correia R Sousa F Vaz A Pedro E Schimidell D . Performance of eight-color dry antibody reagent in the detection of minimal residual disease in chronic lymphocytic leukemia samples. Cytom B Clin Cytom (2020) 98(6):529–35. doi: 10.1002/cyto.b.21875 32251553

[16] Patz M Pentok B Cremer K Linnartz S Lilienweiss E Kleinert F . ROR-1 is a highly discriminative marker in flow cytometric minimal residual disease (MRD) detection in chronic lymphocytic leukemia (CLL). Blood (2016) 128(22):3197–7. doi: 10.1182/blood.V128.22.3197.3197

[17] Farren TW Giustiniani J Fanous M Liu F Macey MG Wright F . Minimal residual disease detection with tumor-specific CD160 correlates with event-free survival in chronic lymphocytic leukemia. Blood Cancer J (2015) 5(1):e273–3. doi: 10.1038/bcj.2014.92 PMC4314455 25615279

[18] D’Arena G Sgambato A Volpe S Coppola G Amodeo R Tirino V . Flow cytometric evaluation of measurable residual disease in chronic lymphocytic leukemia: Where do we stand? Hematol Oncol (2022). doi: 10.1002/hon.3037 35667043

[19] Flores-Montero J Sanoja-Flores L Paiva B Puig N García-Sánchez O Böttcher S . Next generation flow for highly sensitive and standardized detection of minimal residual disease in multiple myeloma. Leukemia (2017) 31(10):2094–103. doi: 10.1038/leu.2017.29 PMC5629369 28104919

[20] Böttcher S Stilgenbauer S Busch R Brüggemann M Raff T Pott C . Standardized MRD flow and ASO IGH RQ-PCR for MRD quantification in CLL patients after rituximab-containing immunochemotherapy: A comparative analysis. Leukemia (2009) 23(11):2007–17. doi: 10.1038/leu.2009.140 19641522

[21] van der Velden VHJ Cazzaniga G Schrauder A Hancock J Bader P Panzer-Grumayer ER . Analysis of minimal residual disease by Ig/TCR gene rearrangements: Guidelines for interpretation of real-time quantitative PCR data. Leukemia (2007) 21(4):604–11. doi: 10.1038/sj.leu.2404586 17287850

[22] Dogliotti I Drandi D Genuardi E Ferrero S . New molecular technologies for minimal residual disease evaluation in b-cell lymphoid malignancies. J Clin Med (2018) 7(9):288. doi: 10.3390/jcm7090288 30231510 PMC6162632

[23] della Starza I Nunes V Cavalli M de Novi LA Ilari C Apicella V . Comparative analysis between RQ-PCR and digital-droplet-PCR of immunoglobulin/T-cell receptor gene rearrangements to monitor minimal residual disease in acute lymphoblastic leukaemia. Br J Haematol (2016) 174(4):541–9. doi: 10.1111/bjh.14082 27172403

[24] Drandi D Kubiczkova-Besse L Ferrero S Dani N Passera R Mantoan B . Minimal residual disease detection by droplet digital PCR in multiple myeloma, mantle cell lymphoma, and follicular lymphoma. J Mol Diagn (2015) 17(6):652–60. doi: 10.1016/j.jmoldx.2015.05.007 26319783

[25] Thompson PA Srivastava J Peterson C Strati P Jorgensen JL Hether T . Minimal residual disease undetectable by next-generation sequencing predicts improved outcome in CLL after chemoimmunotherapy. Blood (2019) 134(22):1951–9. doi: 10.1182/blood.2019001077 PMC6887113 31537528

[26] Wendtner CM . CLL: Deep dive for residual cells by NGS matters. Blood (2019) 134(22):1883–4. doi: 10.1182/blood.2019003244 31778541

[27] Hengeveld PJ van der Klift MY Kolijn PM Davi F Kavelaars FG de Jonge E . Detecting measurable residual disease beyond 10-4 through an IGHV leader-based NGS approach improves prognostic stratification in CLL. Blood (2022). doi: 10.1182/blood.2022017411 36084320

[28] Kotrova M van der Velden VHJ van Dongen JJM Formankova R Sedlacek P Brüggemann M . Next-generation sequencing indicates false-positive MRD results and better predicts prognosis after SCT in patients with childhood ALL. Bone Marrow Transpl (2017) 52(7):962–8. doi: 10.1038/bmt.2017.16 28244980

[29] Efremov DG Laurenti L . Recent advances in the pathogenesis and treatment of chronic lymphocytic leukemia. Pril (Makedon Akad Nauk Umet Odd Med Nauki) (2014) 35(3):105–20. doi: 10.1515/prilozi-2015-0015 25725699

[30] Kwok M Rawstron A Varghese A Evans P O’Connor S Doughty C . Independent prognostic significance of minimal residual disease status in chronic lymphocytic leukaemia. Lancet (2014) 383:S66. doi: 10.1016/S0140-6736(14)60329-9

[31] Abrisqueta P Villamor N Terol MJ González-Barca E González M Ferrà C . Rituximab maintenance after first-line therapy with rituximab, fludarabine, cyclophosphamide, and mitoxantrone (R-FCM) for chronic lymphocytic leukemia. Blood (2013) 122(24):3951–9. doi: 10.1182/blood-2013-05-502773 24124086

[32] Hallek M Fischer K Fingerle-Rowson G Fink A Busch R Mayer J . Addition of rituximab to fludarabine and cyclophosphamide in patients with chronic lymphocytic leukaemia: a randomised, open-label, phase 3 trial. Lancet (2010) 376(9747):1164–74. doi: 10.1016/S0140-6736(10)61381-5 20888994

[33] Goede V Fischer K Busch R Engelke A Eichhorst B Wendtner CM . Obinutuzumab plus chlorambucil in patients with CLL and coexisting conditions. New Engl J Med (2014) 370(12):1101–10. doi: 10.1056/NEJMoa1313984 24401022

[34] Byrd JC Furman RR Coutre SE Flinn IW Burger JA Blum KA . Targeting BTK with ibrutinib in relapsed chronic lymphocytic leukemia. New Engl J Med (2013) 369(1):32–42. doi: 10.1056/NEJMoa1215637 23782158 PMC3772525

[35] Stilgenbauer S Eichhorst B Schetelig J Coutre S Seymour JF Munir T . Venetoclax in relapsed or refractory chronic lymphocytic leukaemia with 17p deletion: a multicentre, open-label, phase 2 study. Lancet Oncol (2016) 17(6):768–78. doi: 10.1016/S1470-2045(16)30019-5 27178240

[36] Heltai S Ghia P Scarfò L . Relevance of minimal residual disease in the era of targeted agents. Cancer J (2019) 25(6):410–7. doi: 10.1097/PPO.0000000000000413 31764122

[37] Uchiyama T Yokoyama A Aoki S . Measurable residual disease in the treatment of chronic lymphocytic leukemia. J Clin Exp Hematop (2020) 60(4):138–45. doi: 10.3960/jslrt.20014 PMC7810249 33148932

[38] Lamanna N Jurcic JG Noy A Maslak P Gencarelli AN Panageas KS . Sequential therapy with fludarabine, high-dose cyclophosphamide, and rituximab in previously untreated patients with chronic lymphocytic leukemia produces high-quality responses: molecular remissions predict for durable complete responses. J Clin Oncol (2009) 27(4):491–7. doi: 10.1200/JCO.2008.16.4459 PMC2645858 19075280

[39] Böttcher S Ritgen M Fischer K Stilgenbauer S Busch RM Fingerle-Rowson G . Minimal residual disease quantification is an independent predictor of progression-free and overall survival in chronic lymphocytic leukemia: A multivariate analysis from the randomized GCLLSG CLL8 trial. J Clin Oncol (2012) 30(9):980–8. doi: 10.1200/JCO.2011.36.9348 22331940

[40] Fischer K Cramer P Busch R Böttcher S Bahlo J Schubert J . Bendamustine in combination with rituximab for previously untreated patients with chronic lymphocytic leukemia: A multicenter phase II trial of the German chronic lymphocytic leukemia study group. J Clin Oncol (2012) 30(26):3209–16. doi: 10.1200/JCO.2011.39.2688 22869884

[41] Burger JA Tedeschi A Barr PM Robak T Owen C Ghia P . Ibrutinib as initial therapy for patients with chronic lymphocytic leukemia. New Engl J Med (2015) 373(25):2425–37. doi: 10.1056/NEJMoa1509388 PMC4722809 26639149

[42] Palma M Mulder TA Österborg A . BTK inhibitors in chronic lymphocytic leukemia: Biological activity and immune effects. Front Immunol (2021) 12:686768. doi: 10.3389/fimmu.2021.686768 34276674 PMC8282344

[43] Ahn IE Farooqui MZH Tian X Valdez J Sun C Soto S. . Depth and durability of response to ibrutinib in CLL: 5-year follow-up of a phase 2 study. Blood (2018) 131(21):2357–66. doi: 10.1182/blood-2017-12-820910 PMC5969380 29483101

[44] Shanafelt TD Wang XV Kay NE Hanson CA O’Brien S Barrientos J . Ibrutinib–rituximab or chemoimmunotherapy for chronic lymphocytic leukemia. New Engl J Med (2019) 381(5):432–43. doi: 10.1056/NEJMoa1817073 PMC6908306 31365801

[45] Fraser G Cramer P Demirkan F Silva RS Grosicki S Pristupa A . Updated results from the phase 3 HELIOS study of ibrutinib, bendamustine, and rituximab in relapsed chronic lymphocytic leukemia/small lymphocytic lymphoma. Leukemia (2019) 33(4):969–80. doi: 10.1038/s41375-018-0276-9 PMC6484712 30315239

[46] Moreno C Greil R Demirkan F Tedeschi A Anz B Larratt L . Ibrutinib plus obinutuzumab versus chlorambucil plus obinutuzumab in first-line treatment of chronic lymphocytic leukaemia (iLLUMINATE): a multicentre, randomised, open-label, phase 3 trial. Lancet Oncol (2019) 20(1):43–56. doi: 10.1016/S1470-2045(18)30788-5 30522969

[47] Lew TE Anderson MA Lin VS Handunnetti SM Came NA Blombery P . Undetectable peripheral blood MRD should be the goal of venetoclax in CLL, but attainment plateaus after 24 months. Blood Adv (2020) 4(1):165–73. doi: 10.1182/bloodadvances.2019000864 PMC6960473 31935286

[48] Mistry H Nduka C Connock M Colquitt J Mantopoulos T Loveman E . Venetoclax for treating chronic lymphocytic leukaemia: An evidence review group perspective of a NICE single technology appraisal. Pharmacoeconomics (2018) 36(4):399–406. doi: 10.1007/s40273-017-0599-9 29222670 PMC5840199

[49] Jones JA Mato AR Wierda WG Davids MS Choi M Cheson BD . Venetoclax for chronic lymphocytic leukaemia progressing after ibrutinib: An interim analysis of a multicentre, open-label, phase 2 trial. Lancet Oncol (2018) 19(1):65–75. doi: 10.1016/S1470-2045(17)30909-9 29246803 PMC6027999

[50] Seymour JF Kipps TJ Eichhorst B Hillmen P D’Rozario J Assouline S . Venetoclax–rituximab in relapsed or refractory chronic lymphocytic leukemia. New Engl J Med (2018) 378(12):1107–20. doi: 10.1056/NEJMoa1713976 29562156

[51] Al-Sawaf O Zhang C Tandon M Sinha A Fink AM Robrecht S . Venetoclax plus obinutuzumab versus chlorambucil plus obinutuzumab for previously untreated chronic lymphocytic leukaemia (CLL14): follow-up results from a multicentre, open-label, randomised, phase 3 trial. Lancet Oncol (2020) 21(9):1188–200. doi: 10.1016/S1470-2045(20)30443-5 32888452

[52] Eichhorst B Niemann C Kater AP Fürstenau M von Tresckow J Zhang C . A randomized phase III study of venetoclax-based time-limited combination treatments (RVe, GVe, GIVe) vs standard chemoimmunotherapy (CIT: FCR/BR) in frontline chronic lymphocytic leukemia (CLL) of fit patients: First Co-primary endpoint analysis of the international intergroup GAIA (CLL13) trial. Blood (2021) 138(Supplement 1):71–1. doi: 10.1182/blood-2021-146161

[53] Wierda WG Allan JN Siddiqi T Kipps TJ Opat S Tedeschi A . Ibrutinib plus venetoclax for first-line treatment of chronic lymphocytic leukemia: Primary analysis results from the minimal residual disease cohort of the randomized phase II CAPTIVATE study. J Clin Oncol (2021) 39(34):3853–65. doi: 10.1200/JCO.21.00807 PMC8713593 34618601

[54] Cervantes-Gomez F Lamothe B Woyach JA Wierda WG Keating MJ Balakrishnan K . Pharmacological and protein profiling suggests venetoclax (ABT-199) as optimal partner with ibrutinib in chronic lymphocytic leukemia. Clin Cancer Res (2015) 21(16):3705–15. doi: 10.1158/1078-0432.CCR-14-2809 PMC4537801 25829398

[55] TamCS AllanJN SiddiqiT KippsTJ JacobsR OpatS . Fixed-duration ibrutinib plus venetoclax for first-line treatment of CLL: Primary analysis of the CAPTIVATE FD cohort. Blood (2022) 139(22):3278–89. doi: 10.1182/blood.2021014488 35196370

